# Influence of Dehydroxymethylepoxyquinomicin on Radiosensitivity of Thyroid Carcinoma TPC-1 Cells

**DOI:** 10.1155/2022/5026308

**Published:** 2022-09-30

**Authors:** Jie Liu, Hu Cai, Heqing Yi, Xin Li, Yunsong Peng, Linfa Li

**Affiliations:** ^1^Department of Nuclear Medicine, Cancer Hospital of the University of Chinese Academy of Sciences (Zhejiang Cancer Hospital), Institute of Basic Medicine and Cancer Chinese, Hangzhou, Zhejiang 310022, China; ^2^Department of Integrated Traditional Chinese and Western Medicine, Cancer Hospital of the University of Chinese Academy of Sciences (Zhejiang Cancer Hospital), Institute of Basic Medicine and Cancer Chinese, Hangzhou, Zhejiang 310022, China; ^3^Department of Pharmacy, Cancer Hospital of the University of Chinese Academy of Sciences (Zhejiang Cancer Hospital), Institute of Basic Medicine and Cancer Chinese, Hangzhou, Zhejiang 310022, China

## Abstract

**Objective:**

To investigate the influence of dehydroxymethylepoxyquinomicin (DHMEQ), an NF-*κ*B inhibitor, on radiosensitivity of thyroid carcinoma (TC) TPC-1 cells.

**Methods:**

The isolation of CDl33 positive cells (CD133^+^ TPC-1) and negative cells (CD133^−^ TPC-1) from TPC-1 cells used immunomagnetic bead sorting. After verification of the toxicity of DHMEQ to cells by MTT and cell cloning assays, the cells were divided into four groups, of which three groups were intervened by DHMEQ, ^131^I radiation, and DHMEQ +^131^I radiation, respectively, while the fourth group was used as a control without treatment. Alterations in cell growth, apoptosis, and cell cycle were observed.

**Results:**

DHMEQ had certain toxic effects on TPC-1 cells, with an IC50 of 38.57 *μ*g/mL (*P* < 0.05). DHMEQ inhibited CD133^+^ and CD133- TPC-1 proliferation and their clonogenesis after irradiation. DHMEQ + radiation contributed to a growth inhibition rate and an apoptosis rate higher than either or them alone (*P* < 0.05), with a more significant effect on CD133^−^ TPC-1 than CD133+ TPC-1 under the same treatment conditions (*P* < 0.05).

**Conclusion:**

DHEMQ can increase the radiosensitivity of TC cells to ^131^I, inhibit tumor cell growth, and promote apoptosis. However, its effect is less significant on CD133^+^ TPC-1 compared with CD133^−^ TPC-1, which may be related to the stem cell-like properties of CD133^+^ cells. In the future, the application of DHMEQ in TC 131I radiotherapy will effectively improve the clinical effect of patients.

## 1. Introduction

Recent years have witnessed the rising incidence of thyroid carcinoma (TC), the most prevalent malignancy of the endocrine system [1]. As indicated by a survey, the global incidence of TC has increased by about 5 times over the past two decades, with a younger age at onset [2]. At present, the accepted standard treatment principle for TC in clinic is the comprehensive therapy based on surgery, supplemented by ^131^I radionuclide therapy and thyrotropin inhibitors [3]. The ten-year survival rate of most TC patients can be as high as 90%-95%, but some still relapse or metastasize after routine treatment, accompanied by radioactive iodine treatment tolerance and reduced ^131^I radiosensitivity, which is called radio-iodine refractory differentiated TC (RR-DTC) [4]. For such patients with a five-year survival rate less than 20%, the treatment difficulty is substantially increased, posing a huge challenge to current TC treatment [5]. Therefore, the clinic is eager to find a new treatment for RR-DTC to improve patients' outcomes.

Nuclear factor-kappa B (NF-*κ*B) is an eukaryotic transcription factor and a highly active molecule that, by binding to DNA, participates in regulating the expression of nearly 400 different genes, realizing the transcription regulation of various genes, cell proliferation, apoptosis, invasion, metastasis, immunity, and other processes [6]. Currently, NF-*κ*B has been confirmed to be activated in many kinds of head and neck tumors including TC, for example, NF-*κ*B-activated miR-574 promotes multiple malignant and metastatic phenotypes by targeting BNIP3 in TC [7]. Whole-genome profiling of nasopharyngeal carcinoma reveals viral-host co-operation in inflammatory NF-*κ*B activation and immune escape [8]. Thus, NF-*κ*B is also considered a breakthrough in future cancer treatment. Dehydroxymethylepoxyquinomicin (DHMEQ) is a novel NF-*κ*B inhibitor developed in recent years and synthesized from quinomycin C, a weak antibiotic [9]. DHMEQ has been shown to suppress NF-*κ*B activity stimulated by bacterial lipopolysaccharide, chemotherapy, radiotherapy, and other stimulating factors. In mice, it inhibited hormone-depleted prostate cancer growth in vivo without side effects [10]. At present, DHMEQ has been found to effectively kill myeloma cells [11] and at the same time reverse the resistance of non-Hodgkin's lymphoma cells to chemotherapy [12], which fully demonstrates the excellent role of DHMEQ in anti-tumor therapy in the future. Furthermore, the research of Pushkarev and Ukaji found that the combined use of paclitaxel and DHMEQ could inhibit the chemotherapy resistance of undifferentiated TC cells to paclitaxel; moreover, compared with common tumor cells, DHMEQ, as an NF-*κ*B inhibitor, is more active in tumor ste0m cells [13, 14]. All these suggest the potential of DHMEQ to improve the treatment status of RR-DTC in the future. However, there is a group of cells with stem cell properties in tumor cells, which has the potential of self-renewal and multi-directional differentiation, which is the basis for tumor occurrence, development, metastasis, recurrence, and anti-radiotherapy and chemotherapy [15]. At present, researchers have found in TC that cancer stem cells are the key cells that cause tumor cell self-renewal and immune escape and reduce the sensitivity of TC cells to chemotherapy or ^131^I [16, 17]. It can be seen that in the research on drug resistance of tumor cells, tumor stem cells are one of the focuses that must be paid attention to. However, although a number of studies have shown the effect of DHMEQ on TC cells and the drug resistance of TC cells [18, 19], no study has yet confirmed whether DHMEQ has the same excellent effect in TC cancer stem cells.

Accordingly, in this experiment, DHMEQ +^131^I radiation was applied to human papillary TC TPC-1 cell line cultured in vitro and isolate its tumor stem cells to observe the changes in their biological behavior, so as to provide new ideas for future treatment of TC and a more reliable guarantee for the prognosis of patients.

## 2. Materials and Methods

### 2.1. Cell Culture

TPC-1 cells of the human papillary TC cell line (purchased from ATCC) were cultured at 37°C with 95% oxygen and 5% carbon dioxide in 10% fetal bovine serum and 1% penicillin/streptomycin-supplemented DMEM (Gibco BRL, Life Technologies, NY, USA). Cells at logarithmic (log) phase were selected for subsequent experiments.

### 2.2. Cell Isolation and Identification

After digestion, log-phase TPC-1 cells (1 × 10^7^) were centrifuged (1000 r/min, 5 min), washed twice with magnetic bead separation buffer, and added with 10 *μ*L CDl33 antibody (designed and constructed by Suzhou Beike Zhenze Biotechnology) with magnetic beads to incubate at 4°C for 15 min. After being cleaned twice with 5 mL buffer, they were immersed in 3 mL buffer and slowly pipetted to form a single-cell suspension, which was then slowly dripped into a CD133 positive separation column placed in a magnetic field in advance, waiting for the natural flow. This was followed by two slow rinses with 3 mL buffer, and buffer (5 mL) addition to the separation tube to quickly wash out CDl33 positive cells (CD133^+^ TPC-1) and negative cells (CD133^−^ TPC-1) adsorbed in the magnetic bead separation tube. Cell purity was verified by flow cytometry (FCM, FACSymphony A1 Flow Cytometer, BD USA) within 30 min and cells were collected for later use and cells after digestion were treated with 5 min of centrifugation at 1000 r/min, two PBS rinses, and addition of 20 *μ*L of fluorescent marker CDl33 antibody, with mouse FITC-IgG2b*κ* antibody (ab136125, Abcam, USA) as a control. After incubating at 4°C for 30 min and washing with buffer twice, the percentage of CDl33^+^ cells was detected by FCM. The differences in the expression of TC stem cell markers OCT-4 and ABCG2 were compared and identified. Identification method: after the addition of protein lysate to verify the purity, the cells were electrophorectically transferred to a PVDF membrane, where they were cultivated at 4°C with OCT4 (1 : 1000, ab265606, Abcam, USA), ABCG2 (1 : 1000, ab207732, Abcam, USA), and *β*-actin (1 : 1000, ab8226, Abcam, USA) primary antibodies for 24 h. The second antibody was added to the membrane the next day, and the gray value was analyzed after ECL (WJ103L, Shanghai Epizyme Biomedical Technology Co., Ltd) development.

### 2.3. Cytotoxicity Test

MTT and cell cloning assays were performed to confirm the optimal treatment dose of DHMEQ, with the method described below.

### 2.4. MTT Assay

Blank, control, and drug groups were set up, with 5 replicate wells in each of them. Log-phase TPC-1 cells (4 × 10^3^/100 *μ*L) were inoculated into the wells of a 96-well cell culture plate. After cell adherence, different concentrations of DHMEQ-containing medium were added. MTT reagent (11465007001, Merck, USA) and DMSO (D2650, Merck, USA) were added, respectively, 24 h after intervention. The absorbance_490 nm_ was measured by ELISA and the IC50 was calculated.

### 2.5. Cell Cloning Assay

Log-phase TPC-1 cells were divided into the following groups for corresponding intervention [20]: ① Blank control group; ② DHMEQ group: only treated with 15 *μ*g/mL DHMEQ; ③ Radiation group: 1Gy irradiation; ④ Joint group: cells were treated with 15 *μ*g/mL DHMEQ for 4 h and then irradiated with 1Gy. After treatment, cell culture was continued for 24 h, and fresh medium was used for routine culture for 7-10 d. The surviving colonies after crystal violet (Y0000418, Merck, USA) staining were observed and microscopically counted. The clone formation rate was calculated for each colony > 50 cells. Clone formation rate (PE%) = number of clones in control group/number of cells in experimental group × 100%.

### 2.6. FCM for Apoptosis

Apoptosis rate: according to the above groupings, cells were treated accordingly. Cells were collected 24 h and 48 h after culture, centrifuged, and washed, and apoptosis was detected by FCM with Annexin V-FITC/PI (APOAF, Merck, USA) double staining. Cell cycle: cells collected after continued culture for 24 h and 48 h were cleaned with PBS and fixed with 70% ethanol. After centrifugation, they were washed twice with PBS at an ambient temperature. They were then treated with 50 *μ*L ribonuclease A, after which propidium iodide was added. Following 15 min of light tight incubation, FCM was used for detection.

### 2.7. Statistical Analysis

SPSS25.0 software processed the data. All assays were repeated 3 times, and the results were expressed as ($\overset{¯}{\chi }\pm s$). The *t*-test was used for between-group comparisons, and analysis of variance and the Tukey-HSD post-hoc test were performed to identify differences among multiple eligible means, with *P* < 0.05 indicating the presence of significance.

## 3. Results

### 3.1. Summary of Results

The results of this experiment found that DHMEQ inhibited CD133^+^ and CD133- TPC-1 proliferation and their clonogenesis after irradiation. DHMEQ + radiation contributed to a growth inhibition rate and an apoptosis rate higher than either or them alone (*P* < 0.05).

### 3.2. Cell Isolation Results

**Figure 1:**
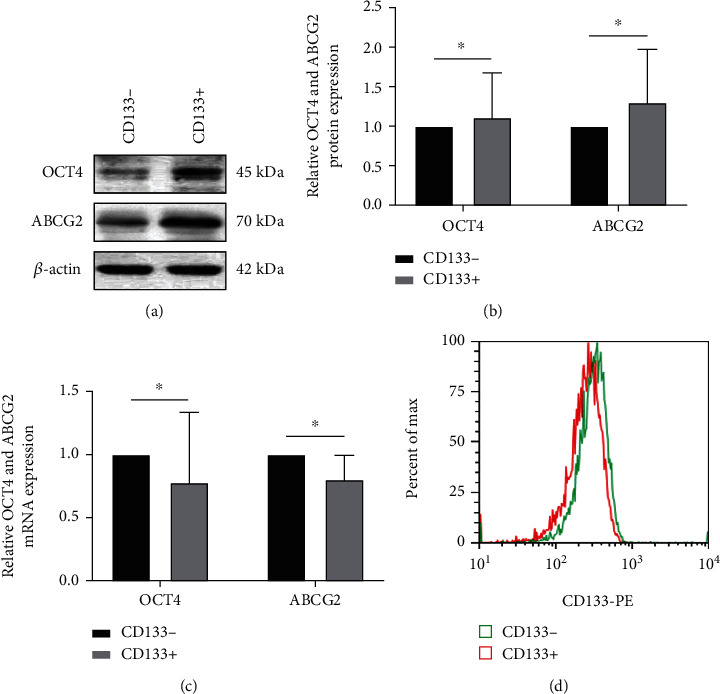
Detection results of OCT4 and ABCG2 protein expression (*n* =3). (a) Western blotting of OCT4 and ABCG2. (b) Expression of OCT4 and ABCG2 protein. (c) Expression of OCT4 and ABCG2 mRNA. (d) CD133 expression detected by flow cytometry. ^∗^*P* < 0.05.

OCT4 and ABCG2 levels were found to be higher in CD133^+^ TPC-1 than in CD133^−^ TPC-1 (*P* < 0.05). FCM analysis showed and identified the deletion of CD133 positivity, indicating successful isolation of stem cells (Figure 1).

### 3.3. Cytotoxicity Test Results

**Figure 2:**
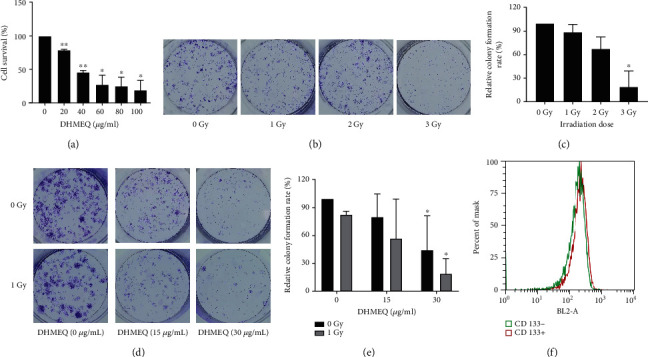
Cytotoxicity test results (*n* =3). (a) MTT assay of cell viability after treatment with different doses of DHMEQ. (b) Cell cloning experiments to detect cell viability after different irradiation doses. (c) Cell cloning rate. (d) Cell cloning assay to detect cell viability after DHMEQ combined with radiation treatment. (e) Cell cloning rate. (f) CD133 expression detected by flow cytometry. ^∗^*P* < 0.05, ^∗∗^*P* < 0.01.

After treating CD133^+^ TPC-1 with different doses of DHMEQ, MTT assay was performed to examine cell growth. It showed that the cell growth capacity of CD133^+^ TPC-1 decreased gradually with the increase of DHMEQ concentration, and the activity reached the lowest when 100 *μ*g/mL DHMEQ was used, with an IC50 of 38.57 *μ*g/mL (*P* < 0.05). Cell cloning assay showed too potent an influence of the irradiation dose of 3Gy on cell viability; instead, 1-Gy irradiation slightly inhibited cell growth ability, which was more suitable for subsequent experiments. Then, DHMEQ with drug concentrations of 0, 15, and 30 *μ*g/mL was used in combination with irradiation doses of 0Gy and 1Gy, respectively. 15 *μ*g/mL DHMEQ plus 1-Gy irradiation was found to have a sensitization effect on cells, and flow cytometry showed that CD133+ TPC-1 cells were positive for CD133, so the optimal dose for subsequent formal experiments was confirmed (Figure 2).

### 3.4. Influence of DHMEQ on Cell Activity

**Figure 3:**
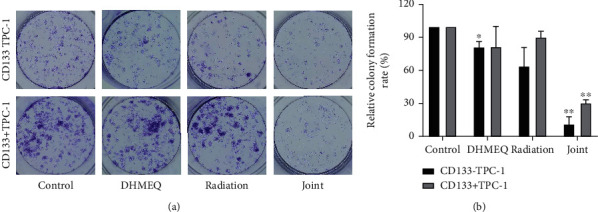
Influence of DHMEQ on cell activity (*n* =3). (a) Results of cell cloning experiment. (b) Cell cloning rate. ^∗^*P* < 0.05, ^∗∗^*P* < 0.01.

Among the four groups of cells, the control group had the strongest colony-forming ability (*P* < 0.05), slightly higher than the DHMEQ group and radiation group (*P* < 0.05). The ability of CD133^+^ TPC-1 and CD133^−^ TPC-1 cells in the joint group to form colonies was the lowest among the four groups (*P* < 0.01). Compared with CD133^−^ TPC-1, the same treatment conditions inhibited the clonogenesis ability of CD133^+^ TPC-1 less (*P* < 0.05) (Figure 3).

### 3.5. Influence of DHMEQ on Apoptosis

**Figure 4:**
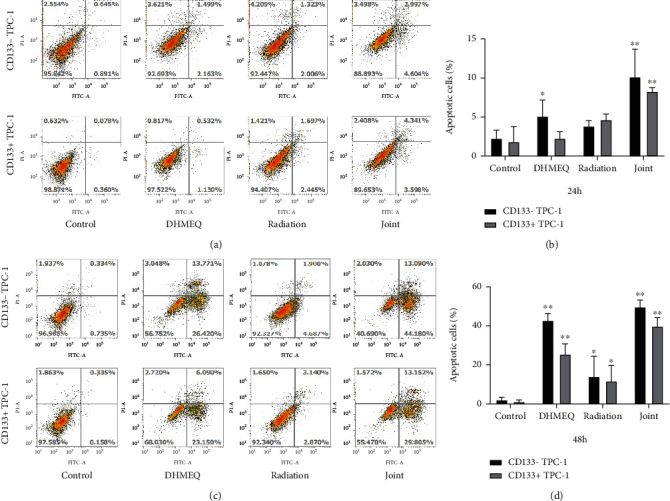
Influence of DHMEQ on apoptosis (*n* =3). (a) Flow cytometry results at 24 h. (b) Flow cytometry results at 48 h. (c) Apoptosis rate at 24 h. (d) Apoptosis rate at 48 h. ^∗^*P* < 0.05, ^∗∗^*P* < 0.01.

FCM analysis results identified that the apoptosis rate of DHMEQ group and control group was the lowest among the four groups at 24 h, while that of the joint group was higher compared with the radiation group (*P* < 0.05). At 48 h, the apoptosis rate was the lowest in the control group among the four groups and the highest in the joint group, while that of the DHMEQ group was higher versus the radiation group (*P* < 0.05). Similarly, under the same conditions, there were fewer apoptotic cells of CD133^+^ TPC-1 (Figure 4).

### 3.6. Impacts of DHMEQ on Cell Cycle

**Figure 5:**
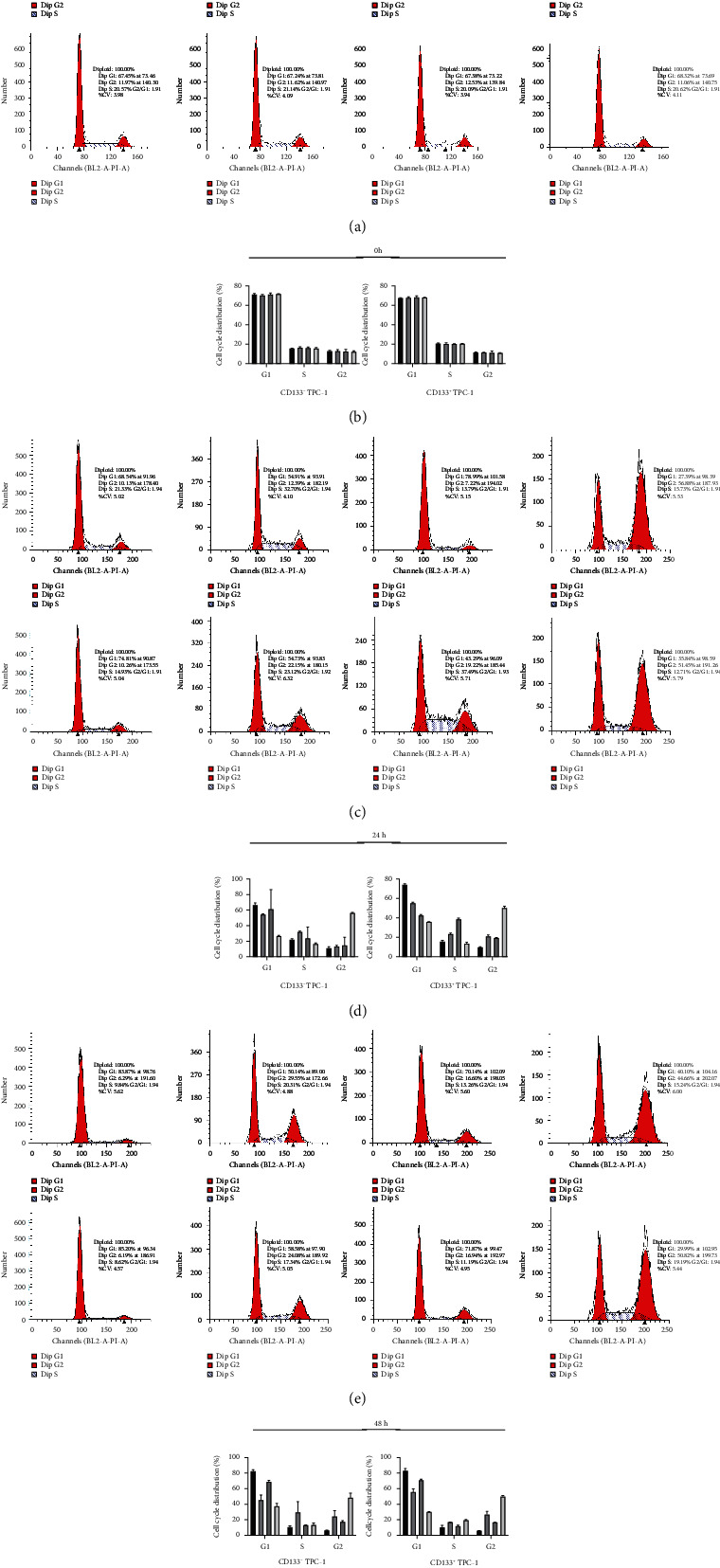
Impact of DHMEQ on cell cycle (*n* =3). (a) Cell cycle distribution at 0 h. (b) Cell cycle changes at 0 h. (c) Cell cycle distribution at 24 h. (d) Cell cycle changes at 24 h. (e) Cell cycle distribution at 48 h. (f) Cell cycle changes at 48 h.

Finally, it can be seen that a large number of cells in the joint group were arrested in the G2 phase. At 24 h and 48 h, the G2-phase cell distribution of CD133^+^ TPC-1 and CD133^−^ TPC-1 in the joint group was higher than that in the other three groups (*P* < 0.05). Thus, DHMEQ + radiation can redistribute the cell cycle in the G2 phase, which is more sensitive to radiation (Figure 5).

## 4. Discussion

Although most TC is difficult to cure, there are still a small number of patients with RR-DTC suffering from adverse prognosis. Hence, it is critical to find effective radiosensitizers [21]. The chemosensitization effect of NF-*κ*B inhibitors on undifferentiated TC cells has been repeatedly demonstrated, which can explain the increasing clinical attention of the involvement of NF-*κ*B pathway in malignant tumor stem cells (including TC stem cells) [22–24]. At present, there is no report about the radiosensitization effect of NF-*κ*B inhibitor DHMEQ on thyroid stem cell-like cells at home and abroad, which may be due to the lack of TC-specific stem cell markers. In the current research, the TPC-1 cell line was selected as the research object by the mature side-population cell sorting method, and the TC side-population cells and non-side-population cells with stem cell characteristics were sorted by FCM. In addition, the sensitivity of DHMEQ to improve ^131^I radiotherapy of differentiated TC side-population cells was evaluated and analyzed by in vitro cell experiments, thus providing new ideas for TC treatment.

First, we isolated TPC-1 stem cells by side-population cell sorting and detected the expression of stem cell protein markers OCT4 and ABCG2. The results showed increased OCT4 and ABCG2 protein expression in CD133^+^ TPC-1 and the deletion of CD133 positivity in CD133^+^ TPC-1, which was consistent with the characteristics of CD133^+^ TPC-1, indicating successful isolation of the stem cells. Second, since there is currently no research on the influence of DHMEQ on TC cells, we first need to confirm its optimal dosage on TC cells. Through the cytotoxicity experiments, it can be seen that under the intervention of 0, 20, 40, 60, 80, and 100 *μ*g/mL of DHMEQ, the cell inhibition rate increased with the increase of DHMEQ concentration, and the IC_50_ of DHMEQ to TPC-1 cells was 38.57 *μ*g/mL, so we further selected 0, 15, and 30 *μ*g/mL for secondary verification. Similarly, among different ^131^I irradiation doses, 3Gy showed the most significant inhibitory effect on cells, but we believe that its activity inhibitory effect is too obvious and may affect the intervention effect of DHEMQ, so 1Gy with lower activity inhibitory effect was selected for follow-up experiments. Under the combined treatment with DHMEQ, the application effect of 15 *μ*g/mL DHMEQ was found to be the most ideal, so we confirm this dose as the final experimental scheme.

Then, through cell cloning and FCM assay, we found inhibited CD133^+^ TPC-1 and CD133^−^ TPC-1 viability and enhanced apoptosis under either DHMEQ intervention or ^131^I irradiation. ^131^I, as one of the current first-line treatment options for TC, has a favorable killing effect on TC cells. The effect of DHMEQ is also consistent with the results of previous studies [25]; it can be seen that the apoptosis rate of cells was significantly increased after the use of DHMEQ, which can confirm the inhibitory effect of DHMEQ on tumor cells. In radiobiology, it is generally believed that G2+M phase cells are most sensitive to radiation, while S-phase cells are the most resistant to radiation [26]. In this study, whether or not CD133 was expressed, TPC-1 cells were more sensitive to radiation after being treated with DHMEQ. After 24 hours of irradiation, the number of CD133^+^ TPC-1 cells in the most radiation-resistant S phase was significantly higher than that of CD133^−^ TPC-1 cells and normal cells and increased with the extension of drug treatment time. And at 48 h, the number of S-phase CD133^−^ TPC-1 of DHMEQ group increased, while the CD133^+^ TPC-1 count decreased. It suggests that DHMEQ plus ^131^I radiotherapy can significantly increase the G2 phase ratio of TPC-1 cells. As we all know, the basic level of NF-*κ*B in tumor cells is higher than that in normal cells, and its main function is to regulate the release of inflammatory factors [27]. Glycolysis in tumor cells produces more Adenosine triphosphate than oxidative phosphorylation, and glucose is an essential nutrient for tumor and normal cell proliferation [28]. Thus, in tumor cells, NF-*κ*B orchestrates many signals of cell activation and proliferation in immune, inflammatory, and carcinogenesis processes [29]. Second, many tumors have activated NF-*κ*B, which keeps cells proliferating and protects them from apoptosis [30]. In addition, the tumor microenvironment often has constitutive NF-*κ*B signaling, which can promote the accumulation of inflammatory factors and tumor-promoting cytokines, further maintaining a favorable environment for tumor growth [31]. In the process of radiotherapy or chemotherapy, NF-*κ*B in tumor cells will be further activated due to the damage of drug toxicity and side effects; high-activity NF-*κ*B can promote the massive production of anti-apoptotic factors, such as Bcl-xL, Bcl-2, IAPs, XIAP, and survivin, and inhibit cancer cell apoptosis, resulting in drug resistance [32]. The increase of NF-*κ*B in TC cells is also related to the occurrence, progression, and chemoradiotherapy tolerance of TC [33]. In the treatment of related tumor diseases, inhibition of NF-*κ*B has become a new generation of therapeutic targets [34]. At present, strategies to inhibit NF-*κ*B signaling mainly include inhibition of protein kinases and phosphatases related to the NF-*κ*B pathway, ubiquitination, acetylation, methylation, and DNA binding steps of NF-*κ*B activity [35]. DHMEQ inhibits its DNA binding by covalently binding to specific cysteine residues of NF-*κ*B components and simultaneously inhibits the activation of macrophages and the maturation of dendritic cells [36]. In this study, DHMEQ induces cancer cell apoptosis through the suppression of NF-*κ*B and can increase the sensitivity of tumor to chemotherapy, which may also be the direct mechanism by which DHMEQ enhances ^131^I radiosensitivity. Of course, more experiments are needed to confirm this. In this study, we can also see that under the same treatment conditions, the effect of DHMEQ on CD133^+^ TPC-1 was not as significant as that on CD133-TPC-1. This may be related to the stem-like properties of CD133^+^ cells, indicating that CD133^+^ cells have a stronger carcinogenic ability, which is consistent with previous studies [37, 38].

However, due to the limited experimental conditions, we were unable to analyze the mechanism of the effect of DHMEQ on TC cells. Therefore, we need to further analyze the mechanism of DHMEQ's effect on TC cells in subsequent studies. In addition, we should also add other TC cell lines for validation experiments to further confirm the effect of DHMEQ on TC cancer stem cells, and verify the effect of DHMEQ on living tumor through nude mouse tumorigenesis experiment, so as to lay a reliable foundation for the future clinical application of DHMEQ, so as to realize the clinical use of DHMEQ and improve the treatment effect and prognosis of TC patients.

## 5. Conclusion

DHEMQ can increase the radiosensitivity of TC cells to ^131^I, inhibit tumor cell growth, and promote apoptosis. However, its effect is less significant on CD133^+^ TPC-1 compared with CD133^−^ TPC-1, which may be related to the stem cell-like properties of CD133^+^ cells.

## Data Availability

The datasets used and/or analyzed during the current study are available from the corresponding author on reasonable request.
